# Effect of a Sulfonated Poly(ether sulfone) Additive on the Bubble Point-Permeability Trade-Off of Polyethersulfone Microfiltration Membranes Prepared by Roll-to-Roll Vapor-Induced Phase Separation

**DOI:** 10.3390/ma19194183

**Published:** 2026-09-30

**Authors:** Soyoung Moon, Jaehyun Yoo, Sungryul Park, Min-Ho Lee

**Affiliations:** 1 UMTR Co., Ltd., Ochang, Cheongju 28116, Republic of Korea; sy@umtrbio.com (S.M.); yjh@umtrbio.com (J.Y.); 2 School of Integrative Engineering, Chung-Ang University, 84 Heukseok-Ro, Dongjak-Gu, Seoul 06974, Republic of Korea

**Keywords:** polyethersulfone, sulfonated poly(ether sulfone), microfiltration, bubble point, permeability, vapor-induced phase separation, roll-to-roll processing, hydrophilic additive

## Abstract

Polyethersulfone (PES) microfiltration (MF) membranes are widely used in bioprocessing and water treatment, but their bubble point (BP), which reflects the largest limiting pore, and their pure water permeability (PWP) are usually coupled in opposite directions, so that gains in throughput are paid for by a loss of pore-size control. Most hydrophilic-additive studies on PES have addressed ultrafiltration membranes cast at laboratory scale, and the effect of a sulfonated poly(ether sulfone) (SPES) additive on the BP–PWP trade-off of MF membranes produced on a continuous line has not been reported. Here, an in-house SPES (potassium form; 25 mol% sulfonated hydroquinone in the monomer feed) was substituted for 0.5 and 1.0 wt% of the PES in casting dopes of constant polymer content, and the dopes were processed into flat-sheet MF membranes on a roll-to-roll vapor-induced/non-solvent-induced phase separation (VIPS/NIPS) line, in which the pore structure is generated by delayed demixing followed by leaching of the water-soluble pore formers poly(vinylpyrrolidone) and poly(ethylene glycol). All membrane series retained the expected negative BP–PWP relation, but the SPES-containing membranes occupied a higher-PWP region than the PES-only control at the same BP; at a matched BP of about 2500 mbar, PWP increased by 10% and 17% at 0.5 and 1.0 wt% SPES. The matched-BP membranes had similar sponge-like morphologies and similar hydraulic mean pore radii, whereas porosity and water uptake increased with SPES content. The results indicate that a small SPES loading expands the control-relative BP–PWP performance window of continuously cast PES MF membranes mainly by increasing the water-accessible void volume rather than by enlarging the pores, and identify SPES as a compositional lever that is compatible with existing industrial casting lines.

## 1. Introduction

Polyethersulfone (PES) is widely used for porous separation membranes because it combines high thermal and hydrolytic stability, mechanical strength, and chemical resistance with established industrial processability [1,2]. In the MF range, PES membranes are used for water treatment, food and beverage clarification, particle removal, and biopharmaceutical processing, where they serve as prefilters and sterilizing-grade or sterilizing-support filters for media, buffers, and product streams [3,4,5]. For these applications, the membrane must combine high hydraulic throughput with a reproducible pore structure, because filtration productivity and retention reliability are both central to process performance.

A persistent limitation of PES is its intrinsic hydrophobicity, which can reduce wetting, lower water permeability, and increase fouling susceptibility [1,2]. Hydrophilic modification of PES membranes has therefore been pursued through bulk modification, surface modification, and additive blending [1,2]. Among these approaches, dope blending is particularly attractive for industrial membrane manufacturing because it can be implemented directly within the casting solution and does not require a separate post-treatment step. Dope blending nevertheless has well-documented limitations. Water-soluble additives such as PVP and PEG are partly leached into the coagulation bath and can continue to leach during operation, so that the hydrophilicity they confer is not necessarily permanent [2,6,7,8]; every additive also changes the dope viscosity and the demixing kinetics, so that its effect on surface chemistry is entangled with its effect on pore structure; and limited miscibility between the additive and the matrix polymer can cause macrophase separation or film defects, which becomes more critical on a continuous casting line than in laboratory casting [2,9].

PES MF and ultrafiltration (UF) membranes are commonly fabricated by phase inversion, in which a homogeneous polymer solution is converted into a porous solid by controlled demixing [10,11,12,13,14,15,16,17,18,19]. In non-solvent-induced phase separation (NIPS), immersion of the cast film into a coagulation bath triggers solvent–nonsolvent exchange and polymer precipitation. The final morphology is governed by the thermodynamic stability of the casting solution and the kinetics of mass transfer: rapid demixing tends to generate finger-like macrovoids, whereas delayed demixing promotes sponge-like, interconnected pore networks [14,15,16,17]. Additives such as poly(vinylpyrrolidone) (PVP), poly(ethylene glycol) (PEG), inorganic salts, and hydrophilic nanoparticles are therefore commonly used to tune demixing, porosity, and surface wetting [2,17,20,21,22,23].

Polymeric additives that are chemically compatible with PES are especially useful because they may reduce macrophase separation while introducing hydrophilic functionality. Sulfonated aryl-sulfone polymers meet this requirement. Sulfonated poly(ether sulfone) and sulfonated polysulfone retain aromatic ether-sulfone backbones related to PES, while sulfonate groups provide ionic and hydrophilic character [24,25,26,27]. Rahimpour et al. showed that blending SPES into PES casting solutions changed membrane morphology, hydrophilicity, and permeability, and Kim and Jung reported that a sulfonated polyethersulfone additive modified the structure and water flux of polysulfone MF membranes [26,27]. Work on PES/sulfonated-polysulfone blends has further shown that a sulfonated aryl-sulfone component can improve compatibility and shift the demixing pathway toward sponge-like asymmetric structures [28,29,30,31]. These studies support the use of sulfonated aryl-sulfone polymers as hydrophilic structural modifiers, but most have focused on static laboratory casting or UF membranes rather than continuous MF membrane processing. Highly sulfonated aryl-sulfone polymers are not used alone as MF membrane materials: the hydrated sulfonate groups increase chain mobility, water uptake, and swelling, which lowers the mechanical strength of the membrane, and their slow precipitation during phase inversion weakens their pore-forming ability [32,33]; they are also considerably more expensive than PES. A small fraction of SPES blended into a PES matrix therefore retains the dimensional stability and processability of PES while introducing sulfonate functionality that is anchored in the matrix through chain entanglement rather than being free to leach [26,34,35].

Vapor-induced phase separation (VIPS) provides an additional handle for morphology control. During VIPS, humid air exposure allows water vapor to diffuse gradually into the cast film before immersion, producing slower nonsolvent uptake than direct coagulation [36,37,38,39,40,41,42,43,44]. This can delay demixing and favor a weakly asymmetric or sponge-like pore network. Coupling VIPS with a subsequent NIPS bath is therefore useful for balancing skin formation, pore connectivity, and film stability. In a roll-to-roll process, this balance is also affected by line speed, dope feed rate, chamber humidity, exposure residence time, and bath temperature; thus, composition and process variables must be considered together, and membranes cast on pilot or production lines can differ appreciably from laboratory-cast membranes of the same composition [9,45].

For MF membranes, BP and PWP are central descriptors. BP is the gas pressure at which the wetting liquid is first displaced from the largest through-pore, or pore throat, of a fully wetted membrane. According to the Young–Laplace relation, BP is proportional to *γ*cos*θ*/*d*_max_, where *γ* is the surface tension of the wetting liquid, *θ* its contact angle on the pore wall, and *d*_max_ the diameter of the largest limiting pore; BP therefore increases as the largest limiting pore becomes smaller and is used in practice as an integrity descriptor of MF membranes [46]. PWP is the volumetric water flux per unit transmembrane pressure. It integrates the hydraulic resistance of the entire water-accessible pore network and depends on the number, size, tortuosity, and connectivity of the pores, on the fraction of pores that are actually wetted, and on the membrane thickness. Increasing pore openness typically increases PWP but lowers BP, while densifying the pore structure raises BP at the expense of PWP. This inverse relation is conceptually related to the permeability-selectivity trade-off that has long been used in membrane science [47,48,49,50]. In the present study, however, this upper-bound concept is used only as a framework for describing coupled membrane performance. The objective is not to define or displace a universal MF upper bound, but to determine whether SPES-containing PES formulations can expand the BP-PWP performance region defined by a PES-only control within a roll-to-roll VIPS/NIPS process window.

Here, an in-house-synthesized SPES additive was blended into PES casting dopes and processed into flat-sheet MF membranes using a continuous roll-to-roll VIPS/NIPS line. Three formulations with increasing SPES content were evaluated by BP, PWP, SEM, surface pore-size analysis, porosity, water uptake, and FT-IR spectroscopy. Because SPES can affect dope hydrophilicity, viscosity, phase-separation behavior, and film stability at the same time, this study was designed as a process-window investigation rather than as a full-factorial single-variable experiment. The central question was whether SPES-containing PES formulations can deliver higher PWP at comparable BP relative to a PES-only control, and whether any gain arises from simple pore enlargement or from increased water-accessible void volume and water-retention capacity.

## 2. Experimental

### 2.1. Materials

Polyethersulfone (PES; Ultrason E 6020 P, BASF, Yeosu, Republic of Korea) was used as the base polymer, with the PES content adjusted within the range of 11.5–12.5 wt%. The hydrophilic polymeric additive was a sulfonated poly(ether sulfone) synthesized in-house and denoted SPES. The monomers used for SPES synthesis were 4,4′-dichlorodiphenyl sulfone (DCDPS, 98%, Haihang Industry, Jinan, China), hydroquinone (HQ, 99%, Samchun Chemical, Pyeongtaek, Republic of Korea), and hydroquinone sulfonic acid potassium salt (sHQ-K, 98.4%, Haihang Industry); potassium carbonate (K_2_CO_3_, 98%, Samchun) was used as the base. 1-Methyl-2-pyrrolidone (NMP, 99.5%, Samchun) and toluene (99.5%, Samchun) were used as the polymerization solvent and azeotroping agent, respectively, and dimethyl sulfoxide (DMSO, >99.5%, Samchun) was used as the casting solvent. Poly(vinylpyrrolidone) K30 (PVP K30, K-value 27–33, Samchun) and poly(ethylene glycol) 200 (PEG 200, Samchun) were used as pore-forming additives. Both are water-soluble: they are largely leached into the coagulation bath and the rinse water and leave behind interconnected pores, and PVP additionally raises the dope viscosity and delays demixing, which suppresses macrovoid formation [2,6,7,21]. PEG 200, a liquid oligomer, also acts as a weak non-solvent that lowers the thermodynamic stability of the dope [21]. Calcium chloride (CaCl_2_, 95%, Junsei Chemical, Tokyo, Japan) was added as an inorganic salt additive; salts such as LiCl and CaCl_2_ interact with the polar solvent and the polymer, increase the dope viscosity, and reduce the thermodynamic stability of the casting solution, which shifts demixing toward sponge-like structures with higher surface porosity [20,51]. Ethanol (99.5%, Samchun) and butanol (99.5%, Samchun) were added to the dope as non-solvent additives that bring the casting solution closer to its binodal so that phase separation is triggered readily during humid-air exposure; they are components of the dope and not of the coagulation bath, which was water (Section 2.3). The dope compositions are summarized in Table 1. SPES was substituted for part of the PES so that the total polymer content was held at 12.5 wt% in all three dopes; the SPES levels of 0.5 and 1.0 wt% of the dope correspond to 4 and 8 wt% of the polymer solids. These low loadings were chosen deliberately. The purpose of this first study was to establish whether the smallest SPES loadings that could be prepared from a single synthesis batch produce a measurable shift of the BP–PWP relation while keeping the dope viscosity and the wet-film stability within the operating window established for the PES-only dope on the continuous line. Higher loadings, at which the water uptake and swelling of the sulfonated component may begin to compromise the mechanical stability of the film [32,33], and the intermediate loadings needed to resolve a dose–response relation were outside the scope of this study and are identified as future work in Section 3.4.

### 2.2. Synthesis of the Sulfonated Poly(ether sulfone) Additive

SPES was synthesized by aromatic nucleophilic substitution polymerization of DCDPS with HQ and sHQ-K. The additive was designed to retain the aromatic ether-sulfone backbone characteristic of PES while incorporating sulfonate groups derived from sHQ-K. In a representative synthesis, DCDPS (1.0 mol), HQ (0.5 mol), and sHQ-K (0.5 mol) were charged into a dried polymerization reactor. On the basis of the total monomer feed, the sHQ-K fraction was approximately 25 mol%. K_2_CO_3_ was added at approximately 1.05 equivalents relative to the total diol content to serve as the base and promote dehydrohalogenation. The monomers and K_2_CO_3_ were dissolved in NMP, dried by azeotropic distillation with toluene, and the reaction was carried out under nitrogen with mechanical stirring at 300 rpm. The mixture was heated to 190 °C and polymerized for 24 h, yielding a viscous polymer solution.

**Figure 1:**
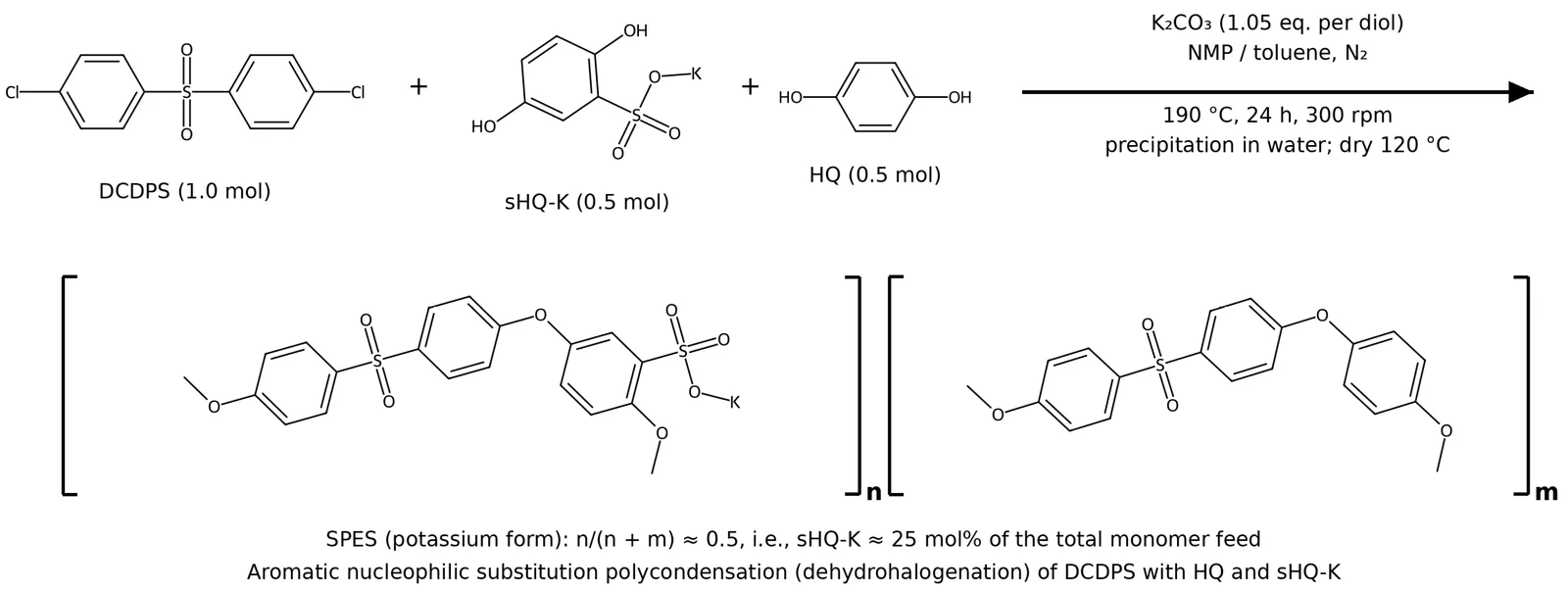
Synthetic route and reaction conditions for the sulfonated poly(ether sulfone) additive (SPES): aromatic nucleophilic substitution polycondensation of DCDPS with HQ and sHQ-K (K_2_CO_3_, NMP/toluene, N_2_, 190 °C, 24 h), giving a potassium-form copolymer with sulfonated (*n*) and non-sulfonated (*m*) ether-sulfone units.

After cooling to room temperature, the polymer solution was precipitated into excess water. The recovered polymer was washed repeatedly with deionized water to remove residual salts, unreacted monomers, and solvent, and then dried overnight at 120 °C. The potassium-form sulfonated poly(ether sulfone) is referred to as SPES throughout this work. The synthetic route is shown in Figure 1. FT-IR and ^1^H NMR spectra of the additive, together with those of its acid and other counter-ion forms, are given in Figure S1 (Supplementary Materials); they show the aromatic ether–sulfone backbone bands of PES with a much stronger O–H band and the aromatic ^1^H NMR signals expected for the sulfonated copolymer.

### 2.3. Membrane Fabrication

**Figure 2:**
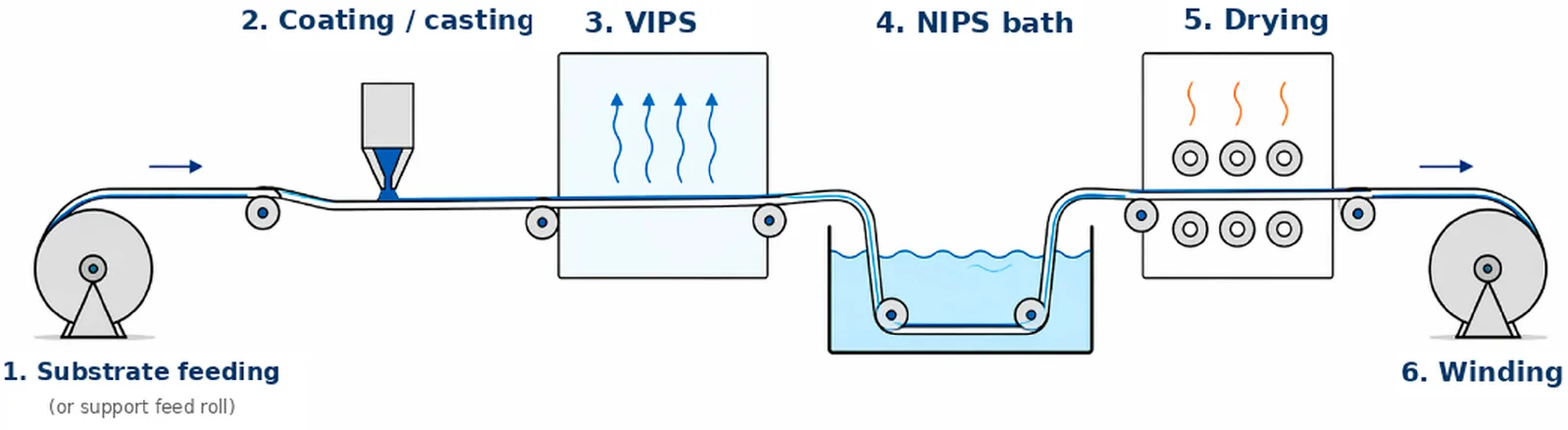
Schematic of the continuous roll-to-roll VIPS/NIPS membrane fabrication process. Horizontal arrows indicate the web travel direction; the wavy blue arrows in the VIPS section represent humidified air, and the orange wavy symbols in the drying section represent heating.

Flat-sheet PES MF membranes were fabricated on a continuous roll-to-roll casting line (Figure 2). The process consisted of substrate feeding, dope coating, a VIPS stage, a NIPS coagulation stage, drying, and winding. The S_0_, S_1_, and S_2_ formulations were not fabricated under a single fixed set of process conditions; instead, the roll-to-roll parameters were adjusted within a defined operating window to obtain stable films and comparable BP regions for each formulation.

The roll speed was varied between 0.25 and 1.0 m/min and the dope feed rate between 42 and 160 mL/min, while the NIPS coagulation bath was maintained at 40 °C for all samples. The VIPS section consisted of four chambers. Different on/off combinations of these chambers were used to adjust humid-air exposure and the initial phase-separation condition of the wet film. All active VIPS chambers were operated at 99% relative humidity. The detailed fabrication conditions are listed in Table 2. In Table 2, the VIPS and air-exposure columns give the residence time of the web in the active (humidified) and inactive chambers of the VIPS section, respectively, and the NIPS column gives the residence time in the coagulation bath; all three follow from the section lengths and the roll speed. The B/A column gives the dope feed per unit web length (feed amount divided by roll speed), which, together with the coating width, sets the wet-film thickness. The dope was applied to the moving web by a slot-die coater at a coating width of 590 mm. The coagulation bath contained water only, at 40 °C; the ethanol and butanol listed in Table 1 are dope components. After coagulation, the membranes were rinsed with water to remove residual solvent and leachable additives, dried, and wound.

This process-window design is important for interpreting the results. Introducing SPES changes the casting formulation and can simultaneously influence dope hydrophilicity, viscosity, solvent–nonsolvent exchange, and wet-film stability. Therefore, the results should be interpreted as the combined composition-process performance of SPES-containing PES formulations within the examined roll-to-roll VIPS/NIPS window, not as the fully isolated single-factor effect of SPES content. The humidity, temperature, and residence time of the VIPS section set the rate and amount of water vapor taken up by the wet film before immersion and thereby the onset of phase separation, the pore size, and the cross-sectional structure. Drying after coagulation removes the water retained in the pore network; because rapid drying causes shrinkage and changes the permeation properties, the drying temperature and time were kept constant for all samples.

### 2.4. Membrane Characterization

Bubble point was measured with a Palltronic Flowstar IV instrument (Pall Corporation, Port Washington, NY, USA) according to the principle of the standard bubble-point method [46]. Circular 47 mm specimens were fully wetted with distilled water, and the pressure at which gas flow was first detected was recorded as the BP. Values are reported in mbar.

Water permeation was evaluated in dead-end filtration mode using a 47 mm circular membrane. The volume of water permeated over 1 min at a constant pressure of 1 bar was measured gravimetrically and converted to volume using a water density of 1 g/mL. PWP was calculated according to Equation (1):

(1) $$\mathrm{PWP}=\frac{V}{A\cdot t\cdot \Delta P}$$

where *V* is the permeate volume, *A* is the effective filtration area, *t* is the filtration time, and Δ*P* is the transmembrane pressure. Because all measurements were performed at 1 bar, the numerical values of flux in LMH and of PWP in LMH/bar are identical, and permeability is reported as PWP throughout.

Membrane porosity was determined gravimetrically from the difference between wet and dry masses. After full wetting, excess surface water was removed, and the wet mass was measured. The membrane was then dried, and the dry mass was measured. Porosity was calculated according to Equation (2):

(2) $$\varepsilon =\frac{W_{\mathrm{wet}}-W_{\mathrm{dry}}}{\rho_{\mathrm{water}}\cdot V_{m}}\times 100$$

where *W*_wet_ and *W*_dry_ are the wet and dry masses of the specimen, *ρ*_water_ is the density of water, and *V*_m_ is the membrane volume obtained from the specimen area and thickness.

The mean pore radius was estimated from the Guerout–Elford–Ferry equation (Equation (3)):

(3) $$r_{m}=\sqrt{\frac{\left(2.9-1.75\varepsilon \right)\cdot 8\eta \mathrm{lQ}}{\varepsilon \cdot A\cdot \Delta P}}$$

where *r*_m_ is the mean pore radius, *ε* is the porosity as a dimensionless fraction, *η* is the viscosity of water, *l* is the membrane thickness, *Q* is the volumetric flow rate, *A* is the filtration area, and Δ*P* is the applied pressure. The calculated values were converted to micrometers. Because this equation uses hydraulic data and porosity as inputs, the resulting pore radius was treated as a comparative hydraulic estimate rather than as an independent structural measurement.

Water uptake was calculated from the wet and dry masses defined above according to Equation (4):

(4) $$\mathrm{Water uptake}=\frac{W_{\mathrm{wet}}-W_{\mathrm{dry}}}{W_{\mathrm{dry}}}\times 100$$

Water uptake was used as a supporting indicator of water-retention capacity that combines the effects of pore volume and of the affinity of the pore walls for water; for a fully saturated membrane it is related to porosity by water uptake = *ε ρ*_water_/[(1 − *ε*) *ρ*_polymer_], so that values well above 100% are expected when the porosity exceeds 70%.

FT-IR spectroscopy was used to compare the chemical features of PES-only and SPES-containing membranes. Spectra were interpreted qualitatively, with particular attention to the broad O-H region near 3500 cm^−1^ and the 1100–1250 cm^−1^ fingerprint region where PES ether/sulfone vibrations and sulfonate-related bands can overlap.

Scanning electron microscopy (SEM) was used to examine surface, cross-sectional, and back-side structures. Cross-sectional specimens were prepared by liquid-nitrogen fracture. Surface and back-side images were recorded at a magnification of 5000× (horizontal field width 25.6 µm) and cross-sections at 400× (horizontal field width 320 µm) at an accelerating voltage of 10 kV; scale bars were added to the images from the calibrated field width. Surface pore-size distributions were obtained from SEM images with an in-house image-analysis tool (SEM/Pore Analysis v4.0; UMTR Co., Ltd., Cheongju, Republic of Korea) and expressed as equivalent circular diameters. Static water contact angles were measured by the sessile-drop method on dry membranes of the S_0_, S_1_, and S_2_ formulations.

### 2.5. Bacterial Challenge and Protein-Adsorption Test Procedures

Bacterial challenge and protein adsorption tests were performed at Inha University on 47 mm discs (effective area 13.8 cm^2^) at 1 bar in dead-end mode, with a commercial 0.22 µm hydrophilic PES membrane (Millipore Express PLUS; Merck Millipore, Burlington, MA, USA) tested under the same conditions as a reference. After measurement of the water permeability and flushing with buffer, 200 mL of a suspension of *Brevundimonas diminuta* (approximately 10^7^ CFU/mL in phosphate-buffered saline, pH 7.4) was filtered, and the permeate was analyzed by plate counting, following the principle of ASTM F838 [52]; the challenge level is reported as CFU per cm^2^ of membrane area and as its logarithm. For protein adsorption, 250 mL of bovine serum albumin (BSA, 1 g/L in phosphate-buffered saline, pH 7.4) was filtered after flushing, and the amount of BSA retained by the membrane was quantified and normalized to the membrane area. The membranes tested were taken from production runs of the PES-only formulation (12.5 wt% PES) and of SPES-containing formulations with 0.5 and 1.0 wt% SPES at 12.0 wt% PES, all prepared with the same SPES synthesis batch and on the same roll-to-roll line as the membranes of Table 2; the last composition differs from S_2_ (Table 1) in its PES content.

## 3. Results and Discussion

### 3.1. BP-PWP Performance Map of SPES-Containing Formulations

**Figure 3:**
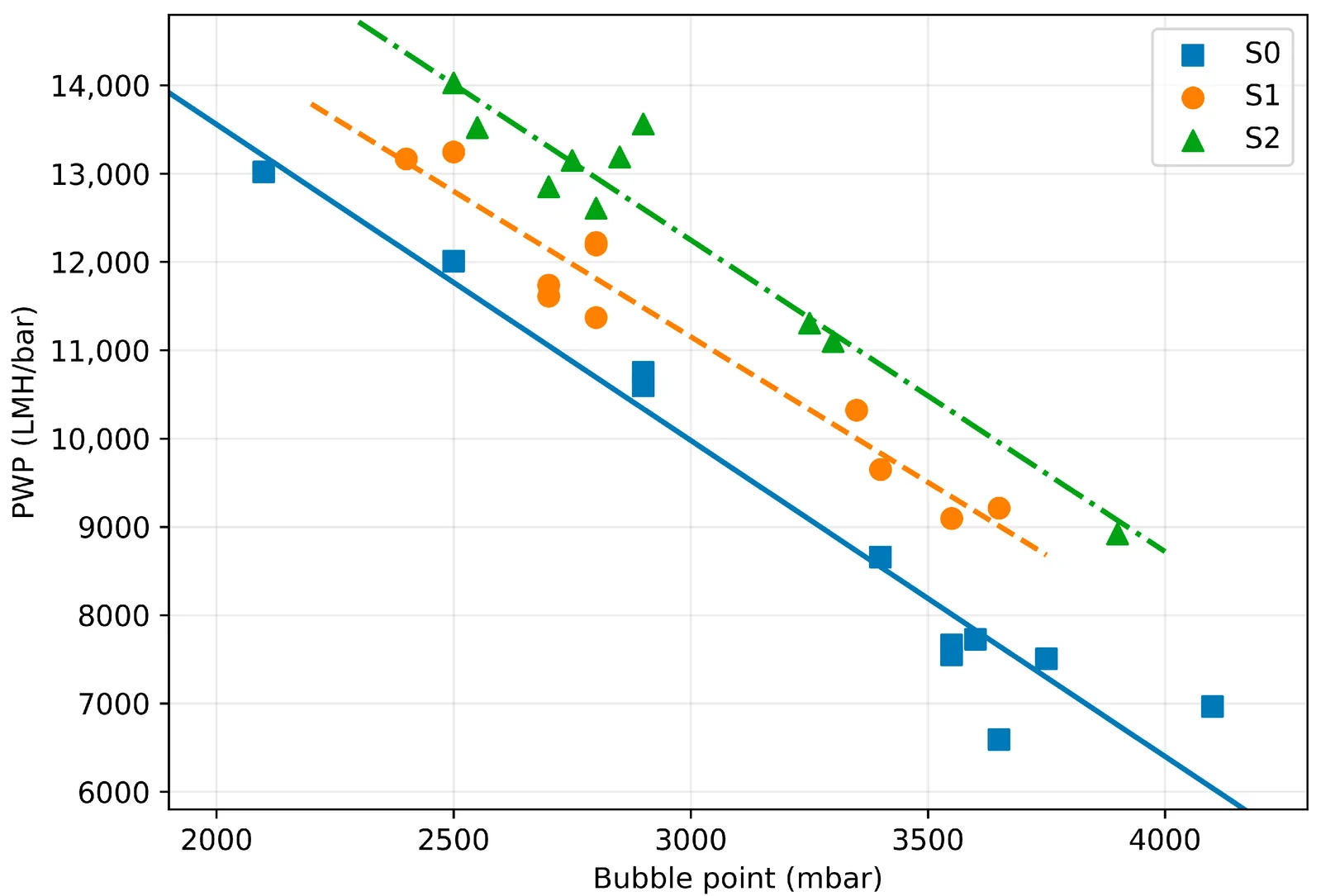
Control-relative BP-PWP performance map of the S_0_, S_1_, and S_2_ membrane series. Linear regression lines are shown for each formulation. All three series retain the expected negative BP-PWP relation, while the SPES-containing S_1_ and S_2_ series occupy a higher-PWP region than the PES-only S_0_ control at comparable BP.

Figure 3 summarizes the BP and PWP of the S_0_, S_1_, and S_2_ membrane series. BP was used as an integrity-related descriptor associated with the largest connected pore or pore throat of the wetted membrane, while PWP was used as the primary hydraulic-performance descriptor [46]. The two quantities are expected to be inversely coupled in MF membranes: opening the pore structure increases water permeability but lowers BP, whereas densifying the pore structure raises BP at the expense of permeability.

The PES-only control series S_0_ followed this expected behavior. As BP increased from 2100 to 4100 mbar, PWP decreased from 13,017 to 6965 LMH/bar. Linear regression of the S_0_ data gave PWP = −3.58BP + 20,717, with R^2^ = 0.944, where PWP is expressed in LMH/bar and BP in mbar. The high coefficient of determination indicates that, within the examined PES-only process window, PWP was strongly governed by the BP-defining pore structure. The S_0_ series therefore provides a suitable control-defined baseline for evaluating whether SPES-containing formulations access a different BP-PWP region.

Several process points in Table 2 were cast more than once at nominally identical settings (S_0_-3 and S_0_-4; S_0_-5, S_0_-6, S_0_-8, S_0_-9, and S_0_-11; S_1_-5 and S_1_-7; S_2_-3, S_2_-4, and S_2_-5; S_2_-6 and S_2_-9). For most of these replicates BP agreed within 100 mbar and PWP within about 10% (S_0_-3/S_0_-4, S_1_-5/S_1_-7, S_2_-3–S_2_-5), whereas some differed more (S_2_-6/S_2_-9; the five S_0_ membranes cast at 0.5 m/min spread over 3400–4100 mbar), which reflects the run-to-run variability of the humid-air stage. This is why the comparison between formulations is based on the fitted BP–PWP relations of the whole series rather than on individual points, and why the matched-BP membranes of Table 3 are characterized by triplicate porosity and water-uptake measurements.

The SPES-containing S_1_ and S_2_ series also retained negative BP-PWP slopes. The fitted relations were PWP = −3.30BP + 21,039 for S_1_ (R^2^ = 0.937) and PWP = −3.53BP + 22,832 for S_2_ (R^2^ = 0.928). Thus, SPES did not remove the general BP-PWP trade-off. Instead, the fitted lines and the individual data points show that the SPES-containing formulations occupied a higher-PWP region than the PES-only control at comparable BP. This distinction is important: the result is not a claim that the intrinsic MF trade-off was eliminated, but that the control-relative performance window was expanded within the examined roll-to-roll VIPS/NIPS process conditions.

The practical significance of this shift is clearest at a matched BP of approximately 2500 mbar. The PWP values of S_0_-2, S_1_-2, and S_2_-1 were 12,007, 13,244, and 14,027 LMH/bar, respectively. Relative to S_0_-2, the corresponding PWP gains were approximately 10.4% for S_1_-2 and 17.1% for S_2_-1. Because the three samples were compared at the same BP, the improvement cannot be described simply as a case of increasing permeability by lowering the BP. Rather, the data indicate higher water transport while maintaining the BP-relevant limiting pore control.

The fitted relationships give the same interpretation across the overlapping BP range. At a fitted BP of 2500 mbar, the predicted PWP values for S_0_, S_1_, and S_2_ were approximately 11,768, 12,800, and 14,012 LMH/bar, respectively. At 3000 mbar, the corresponding values were approximately 9978, 11,152, and 12,248 LMH/bar. These fitted comparisons show that the SPES-containing formulations were not represented by a single outlier, but by a systematic higher-PWP region relative to the control. Because the dataset is limited and was not generated by a full factorial design, the result is described conservatively as control-relative BP-PWP performance-window expansion rather than displacement of a universal MF upper bound.

The absolute PWP level of the present membranes can be placed in context with literature and commercial values, bearing in mind that PWP depends strongly on pore size, thickness, and test conditions. Laboratory-cast PES MF membranes prepared by VIPS-assisted NIPS have been reported with PWP of about 6600–9000 LMH/bar for funnel-like PES membranes [53] and up to 45,700 LMH/bar for a PES-*b*-PEG copolymer membrane with a mean pore diameter of 0.8 µm [54], whereas PES membranes with PVP or PEG pore formers in the ultrafiltration and tight-MF range typically show a few hundred LMH/bar [6,9]. For a commercial 0.22 µm hydrophilic PES membrane (Millipore Express PLUS), the manufacturer specifies a water flow rate of 15 mL min^−1^ cm^−2^ at 0.69 bar, equivalent to roughly 13,000 LMH/bar, at a thickness of 160–185 µm and a bubble point of at least 1.38 bar measured with air/isopropanol [55]. The matched-BP membranes of this study, with PWP of 12,000–14,000 LMH/bar at a water bubble point of 2500 mbar, are therefore in the same range as a sterilizing-grade commercial PES membrane. Such comparisons remain semi-quantitative, however, because the literature values were obtained with different pore sizes, wetting liquids, and pressures; the control-relative comparison within one process window remains the primary evidence of this study.

### 3.2. Matched-BP Structural Comparison

To identify the structural origin of the control-relative PWP increase, the S_0_-2, S_1_-2, and S_2_-1 membranes were selected for matched-BP comparison. Their BP values were all approximately 2500 mbar, while PWP increased from 12,007 to 13,244 and 14,027 LMH/bar (Table 3). This comparison is central to the argument because BP is sensitive to the largest connected pore or pore throat, whereas PWP is an integrated measure of water transport through the entire pore network. If the PWP increase were caused only by the formation of larger limiting pores, a lower BP would be expected. The thicknesses of S_0_-2, S_1_-2, and S_2_-1 were 140, 145, and 146 µm, respectively.

**Figure 4:**
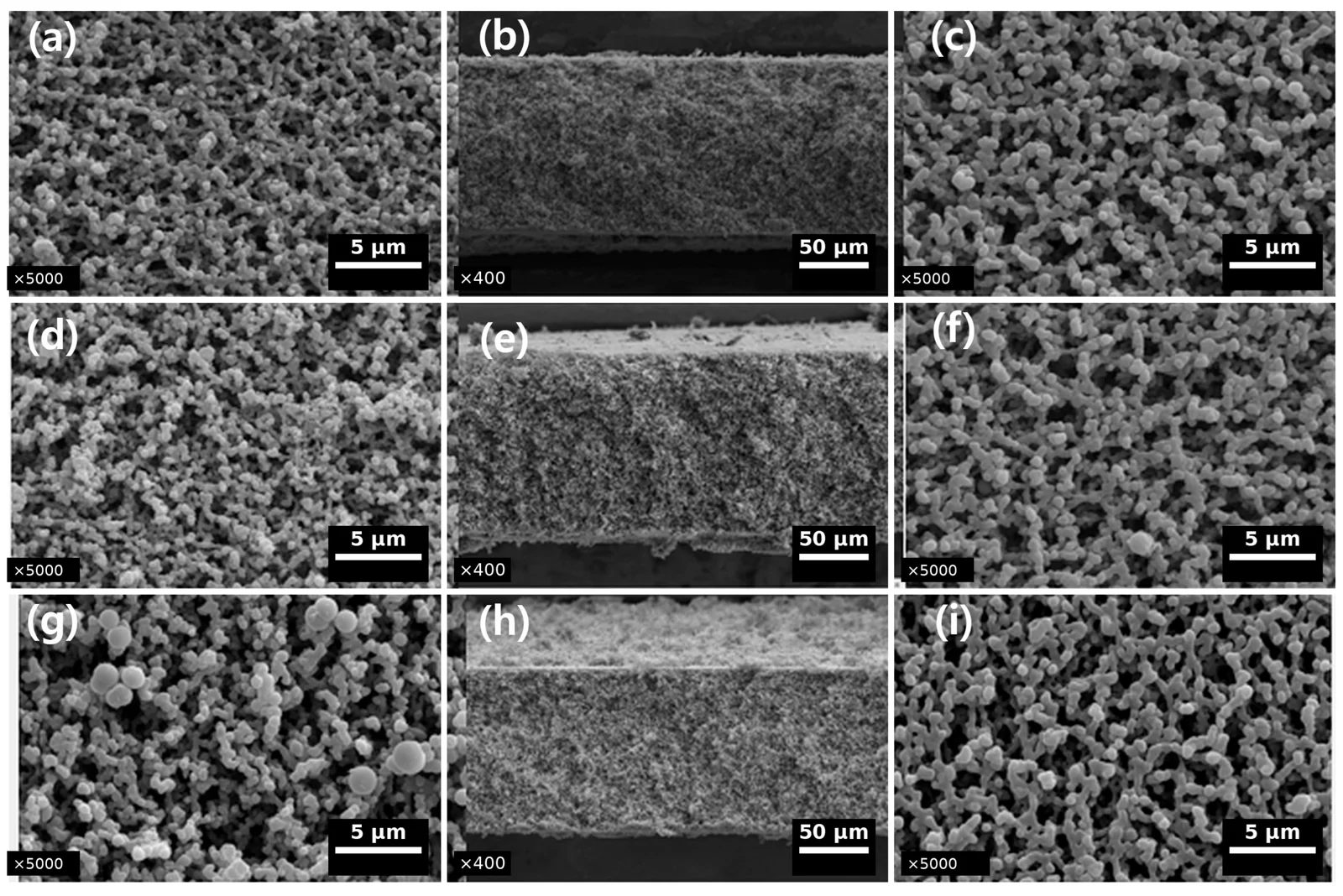
SEM images of the matched-BP membranes: (**a**–**c**) Surface, cross-section, and back side of S_2_-1; (**d**–**f**) surface, cross-section, and back side of S_1_-2; (**g**–**i**) surface, cross-section, and back side of S_0_-2. Surface and back-side images: 5000× (scale bar 5 µm); cross-sections: 400× (scale bar 50 µm).

Figure 4 shows that all three matched-BP membranes had weakly asymmetric, interconnected sponge-like morphologies. A pronounced finger-like macrovoid-dominated structure was not observed in these selected samples. This morphology is consistent with the combined VIPS/NIPS process. During VIPS, gradual water-vapor uptake can delay demixing before immersion, favoring sponge-like porous structures rather than rapid macrovoid formation [36,37,38,39,40,41,42]. Similar delayed-demixing behavior has been reported for PES/sulfonated polysulfone blends, where compatibility between the sulfonated component and PES promotes a sponge-like asymmetric morphology [28,29].

**Figure 5:**
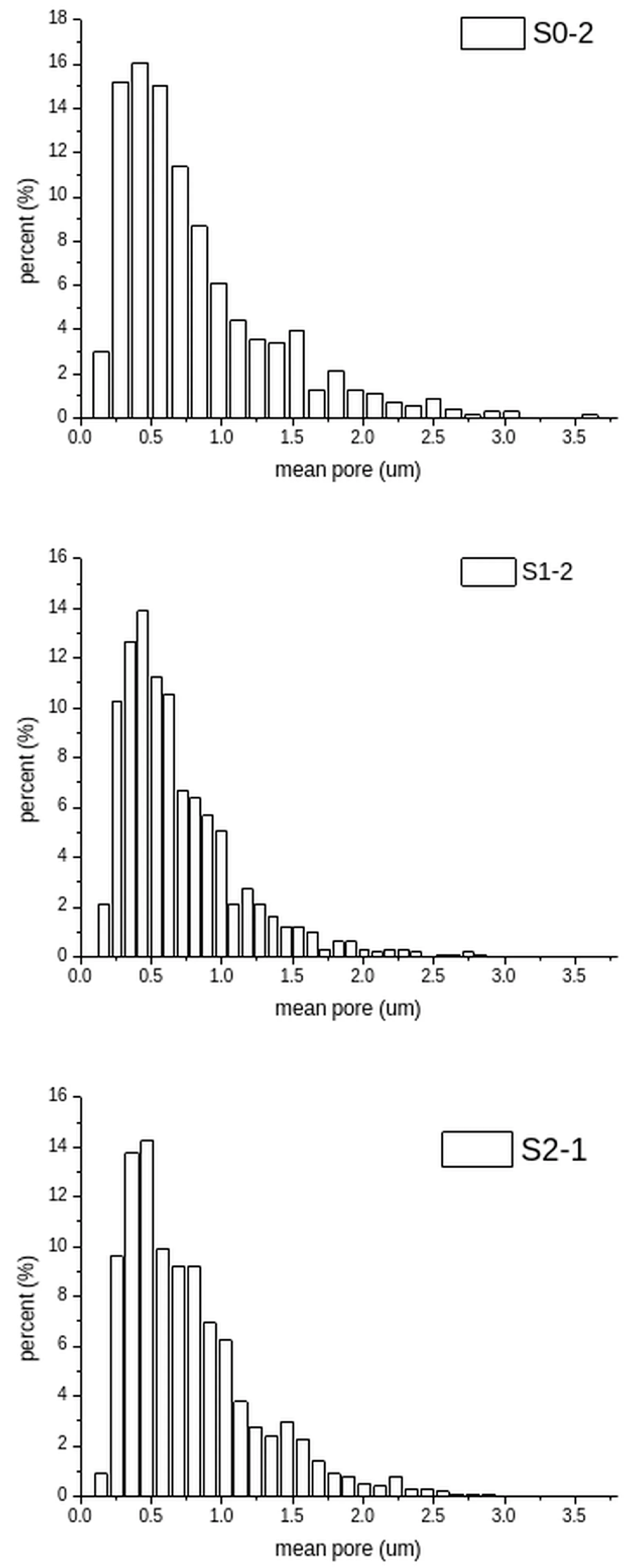
Surface pore-size distributions obtained from SEM image analysis of the matched-BP membranes S_0_-2, S_1_-2, and S_2_-1.

The surface pore-size distributions in Figure 5 support the same interpretation. The principal pore populations of S_0_-2, S_1_-2, and S_2_-1 were concentrated mainly in the 0.4–0.8 µm region, with no monotonic shift toward a larger pore-size population as SPES content increased. The hydraulic mean pore radii estimated from the Guerout–Elford–Ferry equation were also similar: 0.57 µm for S_0_-2, 0.55 µm for S_1_-2, and 0.55 µm for S_2_-1. These values should not be over-interpreted as independent pore-size measurements because the equation uses permeability, porosity, and thickness as inputs. Nevertheless, the agreement between SEM-based distributions and similar calculated hydraulic radius indicates that the PWP enhancement is not explained by simple mean pore enlargement.

The porosity data provide a more direct explanation. Porosity increased from 74.8% for S_0_-2 to 81.1% for S_1_-2 and 83.1% for S_2_-1. These values correspond to absolute increases of 6.3 and 8.3 percentage points relative to the PES-only control. In porous membranes, PWP depends not only on characteristic pore size but also on the number of transport pathways, void fraction, tortuosity, and connectivity. Therefore, higher porosity can increase hydraulic throughput even when the BP-defining pore throat remains controlled. This interpretation is consistent with earlier studies showing that sulfonated PES or sulfonated polysulfone components can alter phase-separation behavior, morphology, and water permeability in PES-based membranes [26,27,28,29,30,31]. The porosity and water-uptake levels are also consistent with the literature on sponge-like PES/sulfonated polymer blend membranes, for which the porosity of PES was reported to rise from 54% to 85% upon blending with a sulfonated polysulfone [34], and with the high bulk porosity of PEG-containing dopes cast on a roll-to-roll line [9].

### 3.3. Porosity and Water Uptake as Water-Transport Descriptors

**Figure 6:**
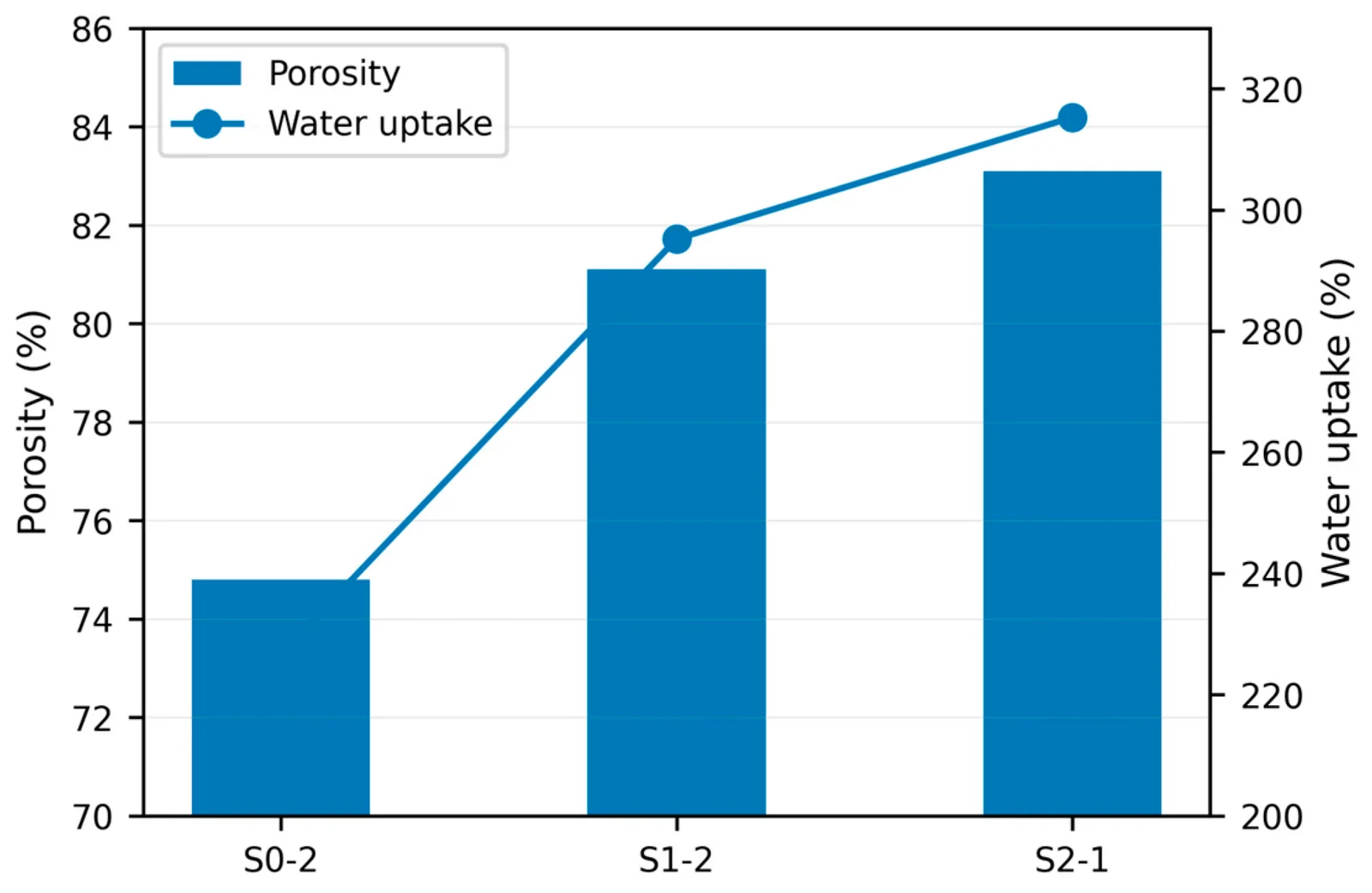
Porosity and water uptake of the matched-BP membranes S_0_-2, S_1_-2, and S_2_-1 (means of three specimens).

The porosity increase was accompanied by a marked increase in water uptake. Water uptake increased from 229.5% for S_0_-2 to 292.3% for S_1_-2 and 315.9% for S_2_-1 (Figure 6). Relative to S_0_-2, these values correspond to increases of approximately 27.4% and 37.7%, respectively. The magnitude and direction of this increase follow the PWP trend more closely than the calculated mean pore radius, supporting the view that the SPES-containing membranes retained more water within a more water-accessible pore network.

An important question is whether the porosity increase alone can account for the PWP gain. For a pore network of similar mean radius, a Hagen–Poiseuille or Kozeny-type description gives PWP ∝ *ε r*_m_^2^/(*τ l*), where *τ* is the tortuosity and *l* the thickness; if *r*_m_ and *τ* are unchanged, PWP scales with *ε*/*l*. The porosity ratios of S_1_-2 and S_2_-1 relative to S_0_-2 are 1.08 and 1.11, their thickness ratios 1.04 and 1.04 (140, 145, and 146 µm), and the measured PWP ratios 1.10 and 1.17. Porosity alone would account for about 80% of the PWP gain of S_1_-2 and about 65% of that of S_2_-1; because the SPES membranes are slightly thicker, the combined porosity–thickness term accounts for roughly 45% and 40%, and the remainder must arise from a lower tortuosity or a better-connected network, which the sponge-like morphologies in Figure 4 cannot resolve, or from more complete wetting of the pore walls, which increases the fraction of the pore network that actually conducts water. The present data cannot separate these contributions; the contact angle data of Section 3.4 argue against a wettability contribution, so the PWP gain is attributed to the larger water-accessible void volume and to the connectivity of the pore network rather than to the hydrophilicity of SPES as such.

Water uptake is likewise not a direct measure of surface hydrophilicity. It reflects the combined effects of pore volume, pore-wall affinity for water, and the ability of the network to retain water after wetting. For a fully saturated membrane, water uptake is fixed by the porosity and the polymer density (Section 2.4): with a PES density of 1.37 g/cm^3^, porosities of 74.8, 81.1, and 83.1% correspond to saturated water uptakes of approximately 217, 313, and 359%. The measured values of 229.5, 292.3, and 315.9% (means of three specimens) lie within about 6–7% of these estimates for S_0_-2 and S_1_-2 and about 12% below the saturation value for S_2_-1, which suggests that a part of the void volume of the most porous membrane was not retained as water at the time of weighing. The water-uptake data are thus consistent with the porosity data and do not by themselves demonstrate a chemical contribution of the sulfonate groups; such a contribution is plausible [24,25,26,27,34] but requires direct wettability measurements, which are discussed in Section 3.4.

The comparison between S_1_-2 and S_2_-1 is informative (Figure 6). Both membranes had the same calculated mean pore radius of 0.55 µm, but S_2_-1 showed higher porosity, higher water uptake, and higher PWP than S_1_-2. This indicates that, within the SPES-containing samples, permeability was governed more strongly by effective void volume, pore-network connectivity, and water retention than by mean pore radius alone. Such behavior is reasonable for sponge-like MF membranes, in which hydraulic resistance is distributed across constrictions, tortuous pathways, and pore-wall wetting rather than determined by a single average pore-size descriptor.

This also explains how BP and PWP can improve simultaneously relative to the PES-only control. BP is governed by the limiting connected pore or pore throat, whereas PWP is an integrated transport property of the full water-accessible network. A membrane can therefore show higher PWP at similar BP if the number or connectivity of transport pathways increases while the largest BP-defining pore throat remains controlled. The matched-BP data for S_0_-2, S_1_-2, and S_2_-1 are consistent with this scenario.

### 3.4. FT-IR Support and Overall Mechanism

**Figure 7:**
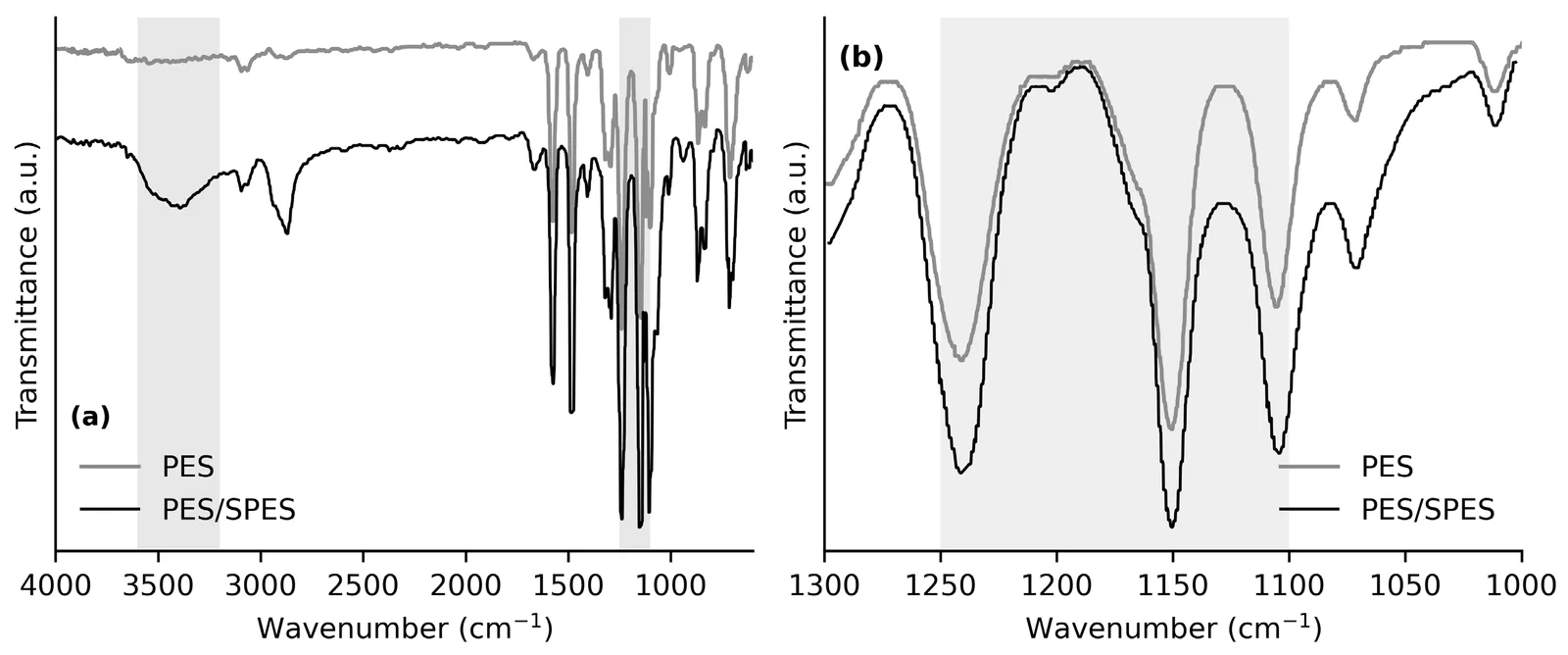
FT-IR spectra of the PES-only and SPES-containing membranes: (**a**) full spectral range (spectra offset vertically for clarity) and (**b**) expanded 1000–1300 cm^−1^ fingerprint region. Shaded bands mark the O–H stretching region near 3500 cm^−1^ and the 1100–1250 cm^−1^ region discussed in the text.

FT-IR spectra were used to examine whether the SPES-containing membrane showed chemical features consistent with sulfonated segments. As shown in Figure 7, both PES-only and SPES-containing membranes retained the characteristic aromatic ether-sulfone backbone signals expected for PES-type polymers. This is consistent with the design of SPES, which retains an aromatic ether-sulfone backbone while introducing sulfonate functionality.

Two regions showed the clearest differences. The broad band near 3500 cm^−1^ is generally associated with O-H stretching of hydrogen-bonded or physically adsorbed water. A stronger contribution in this region for the SPES-containing membrane is consistent with the higher water uptake measured for S_1_-2 and S_2_-1. The 1100–1250 cm^−1^ region requires more cautious interpretation because PES itself exhibits sulfone and aromatic ether vibrations in this range, while sulfonated polymers can also show overlapping S=O and S-O contributions from sulfonate groups [24,25,26]. Therefore, the increased absorption near 1100–1170 cm^−1^ is best interpreted as qualitative support for sulfonate-related contributions, not as a quantitative measurement of SPES content.

The combined performance, morphology, porosity, water-uptake, and FT-IR results support the following mechanism. SPES-containing PES formulations do not simply form larger pores. Instead, within the adjusted roll-to-roll VIPS/NIPS process window, they form a more water-accessible porous network with increased void volume and water-retention capacity while preserving comparable BP-relevant limiting pore control. This mechanism is consistent with previous PES/SPES and PES/SPSf studies, but the present work extends the concept to MF-range PES membranes fabricated under continuous roll-to-roll VIPS/NIPS processing [3,26,27,28,29,30,31].

Water contact angles provide a direct test of the hydrophilicity argument (Table 4). The PES-only membrane showed a static water contact angle of 31.0°, and the 0.5 and 1.0 wt% SPES membranes showed 42.9° and 36.5°, respectively. All three values are far below the 70–90° typically reported for pristine PES [2], which reflects the residual hydrophilic pore formers (PVP, PEG) in the membranes and the fact that, on a membrane with 75–83% porosity and micrometer-scale surface pores, the apparent contact angle is governed by surface porosity, roughness, and wicking rather than by the intrinsic surface energy alone. More importantly, the SPES-containing membranes did not show a lower contact angle than the control. The data therefore do not support an increase in surface hydrophilicity at these SPES loadings, and they confirm the interpretation of Section 3.3 that the PWP gain at matched BP arises from the larger water-accessible void volume rather than from a measurable change in wettability. Surface free energy was not determined because two-liquid methods are unreliable on such highly porous, wicking surfaces; contact angles on dense PES/SPES films, which would isolate the intrinsic contribution of the sulfonate groups, are identified as future work.

From a manufacturing standpoint, the SPES route requires no additional process step and no capital equipment: the additive is dissolved in the dope, and the membrane is cast, coagulated, rinsed, and dried exactly as the PES-only membrane. The additional material cost is that 4–8 wt% of the polymer solids is an in-house sulfonated copolymer instead of commodity PES. A 10–17% increase in PWP at unchanged BP translates into a proportionally smaller filter area, or a shorter filtration time, for a given batch at an unchanged integrity specification; because the additive is a small fraction of the membrane cost, this gain is expected to outweigh the additive cost, although a formal cost analysis was not performed and the retention limit at higher loadings (Section 3.5) must be respected.

The limitations of this interpretation should be stated explicitly. First, the roll-to-roll conditions were adjusted for each formulation, so the dataset does not isolate SPES content as the only independent variable. Second, surface pore-size analysis and Guerout–Elford–Ferry estimates do not replace capillary-flow porometry. Third, FT-IR of the membranes and the FT-IR and ^1^H NMR spectra of the additive (Figure S1) confirm the presence of sulfonated units qualitatively but do not quantify the degree of sulfonation of the additive or its enrichment at the membrane surface. Fourth, only two SPES loadings were examined, so the dose–response relation and the upper limit of useful SPES content remain to be established. Fifth, the roll-to-roll line and the in-house additive limit the number of formulations that can be screened, and the matched-BP comparison rests on one membrane per formulation. Future work should include a wider SPES concentration series, contact angle and surface free energy measurements on dense PES and PES/SPES films, capillary-flow porometry, dope viscosity and cloud-point measurements, and SPES ion-exchange capacity to clarify the mechanism, together with bacterial retention testing under ASTM F838 [52] at every SPES loading to extend the retention data of Section 3.5.

### 3.5. Bacterial Retention and Protein Adsorption

Because the intended application of these membranes is bioprocess filtration, their practical performance was assessed by bacterial retention and protein adsorption rather than by a water-purification test (Table 5). These tests were performed on the 0.2 µm base grade from which the present formulations derive; the same measurements also serve, for a different purpose, to verify the retentive base layer of the third-generation composite membranes described in a companion manuscript [56], and they are reported here with that cross-reference rather than as new data. The PES-only membrane and the 0.5 wt% SPES membrane retained the complete challenge of 1.1 × 10^9^ and 4.1 × 10^8^ CFU/cm^2^ of *B. diminuta* (no colonies in 200 mL of permeate; 9.0 and 8.6 log CFU/cm^2^), which is the same level of retention as the commercial 0.22 µm PES membrane tested alongside (8.7 log CFU/cm^2^), at water permeabilities of about 11,100 and 10,100 LMH/bar on the 13.8 cm^2^ test cell. Their BSA adsorption, 5.5 and 10.2 µg/cm^2^, was 0.3 and 0.6 times that of the commercial membrane (18.1 µg/cm^2^). The 1.0 wt% SPES membrane tested in this series (12.0 wt% PES) had the highest permeability (about 12,200 LMH/bar) but did not retain the challenge—colonies in the permeate were too numerous to count—and adsorbed more BSA (22.6 µg/cm^2^) than the commercial membrane. These results show that a small SPES loading (0.5 wt%) preserves sterilizing-grade bacterial retention and low protein binding while increasing permeability, whereas the particular 1.0 wt% membrane tested lost retention. Whether this reflects the higher SPES content or the process point of that production run cannot be decided from a single membrane, because the bubble point of the tested discs was not part of the retention protocol; the observation nevertheless indicates that retention must be verified at each SPES loading and that, until a dose–response study is completed, 0.5 wt% is the loading for which both the permeability gain and the retention have been demonstrated.

## 4. Conclusions

An in-house-synthesized sulfonated poly(ether sulfone) additive was blended into PES casting dope and processed into MF membranes by continuous roll-to-roll VIPS/NIPS. The study was designed as a composition-process window investigation to determine whether SPES-containing PES formulations can deliver higher PWP at comparable BP relative to a PES-only control.

All membrane series followed the expected negative BP-PWP relationship. However, the SPES-containing S_1_ and S_2_ formulations occupied a higher-PWP region than the S_0_ control. At a matched BP of approximately 2500 mbar, PWP increased from 12,007 LMH/bar for S_0_-2 to 13,244 LMH/bar for S_1_-2 and 14,027 LMH/bar for S_2_-1, corresponding to gains of 10.4% and 17.1% relative to the PES-only control.

The matched-BP membranes had similar calculated mean pore radii (0.57, 0.55, and 0.55 µm), and SEM-based pore-size distributions did not show a simple monotonic shift toward larger pores with SPES addition. By contrast, porosity increased from 74.8 to 83.1%, and water uptake increased from 229.5 to 315.9%. FT-IR spectra qualitatively supported sulfonate-related contributions and higher water retention, although they were not treated as quantitative proof of SPES content.

Overall, the results identify SPES as a promising compositional design factor for improving the BP-PWP balance of PES MF membranes. Within the examined roll-to-roll VIPS/NIPS process window, SPES-containing formulations expanded the control-relative BP-PWP performance window by increasing the effective void volume and water-retention capacity rather than by simply enlarging the mean pore size; the porosity increase accounts for a large part of the PWP gain, the remainder being attributed to pore-network connectivity, while the water contact angles give no evidence of a hydrophilicity contribution. Water contact angles did not decrease with SPES, and bacterial challenge tests showed that the 0.5 wt% SPES membrane retains sterilizing-grade bacterial retention and low protein adsorption, whereas retention must be verified at higher loadings. Because the SPES is introduced through the dope, the approach can be implemented on existing industrial casting lines without additional process steps.

## Figures and Tables

**Table 1:** Dope solution compositions of the S_0_–S_2_ membranes.

Sample	PES (wt%)	SPES (wt%)	DMSO (wt%)	CaCl_2_ (wt%)	PVP K30 (wt%)	PEG 200 (wt%)	Ethanol/ Butanol (wt%)
S_0_	12.5	0	46	1	0.5	30	5/5
S_1_	12.0	0.5	46	1	0.5	30	5/5
S_2_	11.5	1.0	46	1	0.5	30	5/5

**Table 2:** Roll-to-roll fabrication conditions for each membrane sample.

Sample	Roll Speed (m/min)	Feed Amount (mL/min)	VIPS (min)	Air Exposure (min)	NIPS (min)	B/A (mL/m)	BP (mbar)	PWP (LMH/Bar)
S_1_-1	0.75	120	1.7	5.2	2.4	160	2400	13,169
S_1_-2	1	160	2.6	2.6	1.8	160	2500	13,244
S_2_-6	0.75	125	3.5	3.5	2.4	166.6	2850	13,190
S_2_-9	0.75	125	3.5	3.5	2.4	166.6	3300	11,101
S_1_-9	0.75	125	3.5	3.5	2.4	166.6	3400	9648
S_2_-1	0.75	125	5.2	1.7	2.4	166.6	2500	14,027
S_2_-10	0.75	125	5.2	1.7	2.4	166.6	3900	8922
S_0_-2	0.25	42	5	15	7	168	2500	12,007
S_0_-3	0.25	42	10	10	7	168	2900	10,596
S_0_-4	0.25	42	10	10	7	168	2900	10,748
S_0_-10	0.25	42	10	10	7	168	3750	7504
S_0_-1	0.25	42	10	10	7	168	2100	13,017
S_2_-2	0.65	110	6	2	2.8	169.2	2550	13,522
S_1_-7	0.65	110	4	4	2.8	169.2	2800	12,194
S_1_-5	0.65	110	4	4	2.8	169.2	2800	11,371
S_1_-3	0.65	110	4	4	2.8	169.2	2700	11,613
S_0_-5	0.5	85	2.5	7.5	3.5	170	3400	8659
S_0_-6	0.5	85	2.5	7.5	3.5	170	3550	7546
S_0_-8	0.5	85	2.5	7.5	3.5	170	3600	7725
S_0_-9	0.5	85	2.5	7.5	3.5	170	3650	6591
S_0_-11	0.5	85	2.5	7.5	3.5	170	4100	6965
S_1_-4	0.5	85	2.6	7.8	3.5	170	2700	11,737
S_1_-6	0.5	85	2.6	7.8	3.5	170	2800	12,221
S_2_-3	0.5	85	5	5	3.5	170	2700	12,851
S_2_-4	0.5	85	5	5	3.5	170	2750	13,148
S_2_-5	0.5	85	5	5	3.5	170	2800	12,609
S_1_-11	0.7	125	3.7	3.7	2.6	178.5	3650	9213
S_1_-10	0.75	135	3.5	3.5	2.4	180	3550	9095
S_1_-8	0.8	145	3.3	3.3	2.3	181.2	3350	10,323
S_2_-7	0.65	125	4	4	2.8	192.3	2900	13,563
S_2_-8	0.65	125	4	4	2.8	192.3	3250	11,308
S_0_-7	0.5	105	2.5	7.5	3.5	210	3550	7663

VIPS, residence time in the active humid-air chambers; air exposure, residence time in the inactive chambers of the VIPS section; NIPS, residence time in the coagulation bath; B/A, dope feed per unit web length (feed amount/roll speed). BP and PWP are the measured bubble point and pure water permeability of the resulting membrane (1 bar, 47 mm specimens).

**Table 3:** Performance and structure descriptors of the matched-BP membranes. Porosity and water uptake are means ± standard deviations of three specimens; thicknesses 140, 145, and 146 µm.

Sample	BP (mbar)	PWP (LMH/Bar)	Porosity (%)	Water Uptake (%)	r_m_ (µm)
S_0_-2	2500	12,007	74.8 ± 2.7	229.5 ± 27.2	0.57
S_1_-2	2500	13,244	81.1 ± 2.6	292.3 ± 36.5	0.55
S_2_-1	2500	14,027	83.1 ± 2.7	315.9 ± 22.9	0.55

**Table 4:** Static water contact angles of membranes of the S_0_, S_1_, and S_2_ formulations.

Formulation	PES (wt%)	SPES (wt%)	Water Contact Angle (°)
S_0_	12.5	0	31.0
S_1_	12.0	0.5	42.9
S_2_	11.5	1.0	36.5

Sessile-drop method on dry membranes from separate production runs of the same formulations.

**Table 5:** Bacterial retention and BSA adsorption of PES-only and SPES-containing membranes and of a commercial 0.22 µm PES membrane (13.8 cm^2^, 1 bar).

Membrane	Water Permeability (LMH/Bar)	Feed (CFU/mL)	Permeate (CFU/mL)	Challenge (CFU/cm^2^)	Challenge (log CFU/cm^2^)	BSA Adsorption (µg/cm^2^)
PES only (12.5 wt%)	≈11,100	7.7 × 10^7^	0	1.1 × 10^9^	9.0	5.5
0.5 wt% SPES (12.0 wt% PES)	≈10,100	2.9 × 10^7^	0	4.1 × 10^8^	8.6	10.2
1.0 wt% SPES (12.0 wt% PES)	≈12,200	9.3 × 10^7^	TMTC	1.3 × 10^9^	9.1	22.6
Millipore Express PLUS 0.22 µm	≈11,400	4.0 × 10^7^	7.6	5.8 × 10^8^	8.7	18.1

Challenge, total CFU applied per membrane area (200 mL permeate); TMTC, too many to count. Tests performed at Inha University on membranes from production runs of the same formulations, not on the matched-BP membranes of Table 3. The PES-only, 0.5 wt% and 1.0 wt% rows are also reported in the companion manuscript [56], where they verify the base layer of composite membranes.

## Data Availability

The original contributions presented in this study are included in the article/Supplementary Materials. Further inquiries can be directed to the corresponding authors.

## Supplementary Materials

The following supporting information can be downloaded at: https://www.mdpi.com/article/10.3390/ma19194183/s1, Figure S1: FT-IR and ^1^H NMR spectra of the sulfonated poly(ether sulfone) additive.
