# Supplementary material for: Effect of a Sulfonated Poly(ether sulfone) Additive on the Bubble Point-Permeability Trade-Off of Polyethersulfone Microfiltration Membranes Prepared by Roll-to-Roll Vapor-Induced Phase Separation
Source: Materials (Basel). 2026 Sep 30;19(19):4183. doi: 10.3390/ma19194183 (PMC13666017; doi:10.3390/ma19194183)
Supplement: Supplementary file 1 [file materials-19-04183-s001.zip › materials-4571307-supplementary.pdf]

Supplementary Materials

Effect of a Sulfonated Poly(ether sulfone) Additive on the Bubble Point–Permeability Trade-Off of Polyethersulfone Microfiltration Membranes Prepared by Roll-to-Roll Vapor-Induced Phase Separation

Soyoung Moon, Jaehyun Yoo, Sungryul Park* and Min-Ho Lee*

(a)

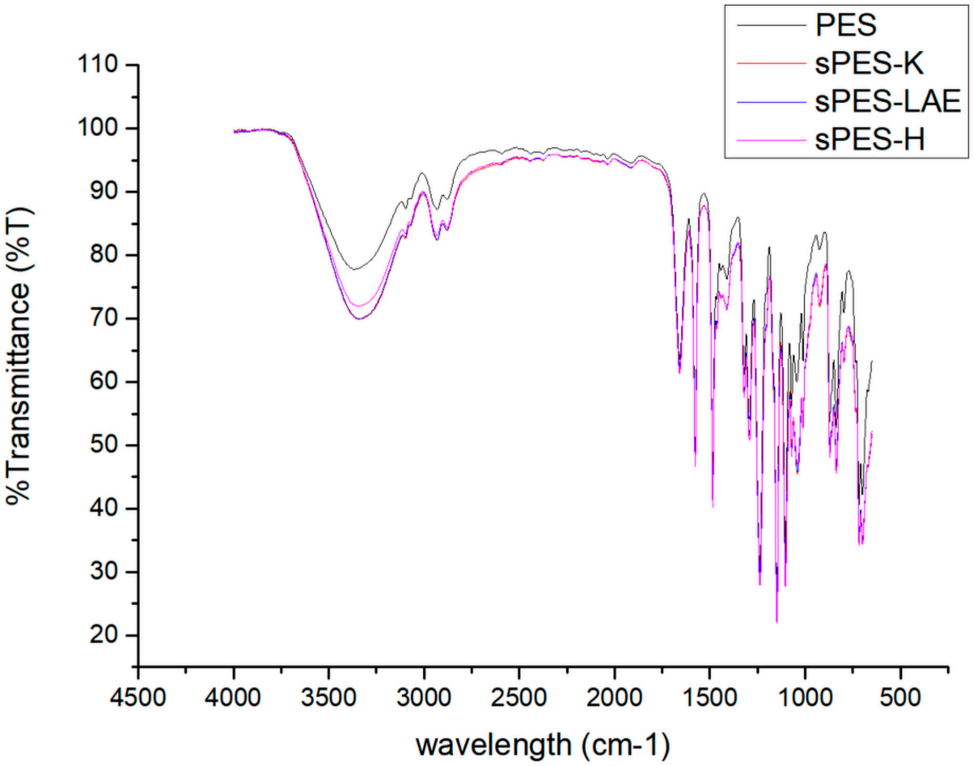
PES
sPES-K
sPES-LAE
sPES-H
%Transmittance (%T)
110
100
90
80
70
60
50
40
30
20
4500
4000
3500
3000
2500
2000
1500
1000
500
wavelength (cm-1)

(b)

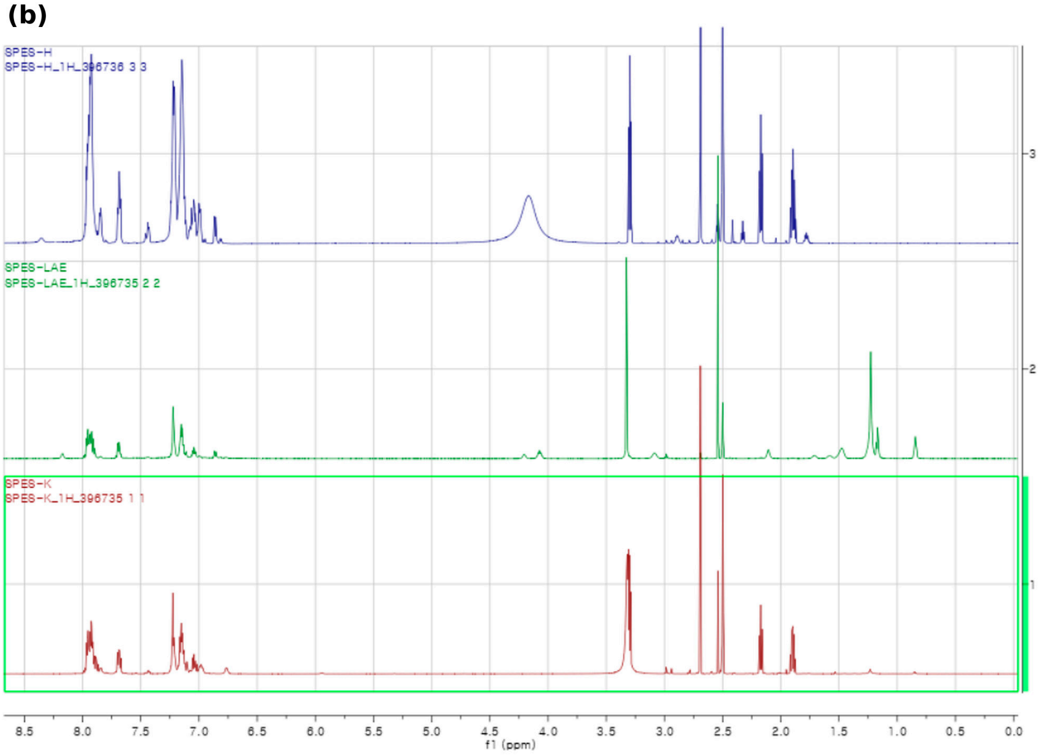
SPES-H
SPES-LAE
SPES-K
8.5
8.0
7.5
7.0
6.5
6.0
5.5
5.0
4.5
4.0
3.5
3.0
2.5
2.0
1.5
1.0
0.5
0.0
f1 (ppm)

Figure S1. Characterization of the synthesized sulfonated poly(ether sulfone) additive. (a) FT-IR spectra (transmittance) of PES powder and of the sulfonated poly(ether sulfone) in its potassium form (sPES-K, the SPES additive used in this work) and in two other counter-ion forms prepared from the same polymer (sPES-H, acid form; sPES-LAE). The sulfonated polymers retain the aromatic ether–sulfone backbone bands of PES and show a markedly stronger broad O–H band near 3300–3400 cm^{-1}, consistent with the hygroscopic sulfonate groups. (b) ^{1}H NMR spectra (DMSO-d6) of sPES-K, sPES-LAE and sPES-H, showing the aromatic multiplets of the copolymer between 6.8 and 8.1 ppm, including the downfield signals near 7.9–8.1 ppm assigned to the aromatic proton ortho to the sulfonate group of the sulfonated hydroquinone unit; the signals near 1.9, 2.2 and 3.3 ppm are residual N-methyl-2-pyrrolidone. The spectra are shown as recorded and are used as qualitative evidence of sulfonation; the degree of sulfonation was not quantified.
